# Improving Genomic Predictions in Multi-Breed Cattle Populations: A Comparative Analysis of BayesR and GBLUP Models

**DOI:** 10.3390/genes15020253

**Published:** 2024-02-18

**Authors:** Haoran Ma, Hongwei Li, Fei Ge, Huqiong Zhao, Bo Zhu, Lupei Zhang, Huijiang Gao, Lingyang Xu, Junya Li, Zezhao Wang

**Affiliations:** 1 Institute of Animal Sciences, Chinese Academy of Agricultural Sciences, Beijing 100193, China; mahaorancaas@163.com (H.M.); hongwei9@ualberta.ca (H.L.); zhanglupei@caas.cn (L.Z.); lijunya@caas.cn (J.L.); 2 Department of Agricultural, Food and Nutritional Science, University of Alberta, Edmonton, AB 510632, Canada; 3 College of Animal Science, Shanxi Agricultural University, Jinzhong 030801, China

**Keywords:** genomic prediction, multi-breed prediction, weighted G-matrix, BayesR, prediction accuracy

## Abstract

Numerous studies have shown that combining populations from similar or closely related genetic breeds improves the accuracy of genomic predictions (GP). Extensive experimentation with diverse Bayesian and genomic best linear unbiased prediction (GBLUP) models have been developed to explore multi-breed genomic selection (GS) in livestock, ultimately establishing them as successful approaches for predicting genomic estimated breeding value (GEBV). This study aimed to assess the effectiveness of using BayesR and GBLUP models with linkage disequilibrium (LD)-weighted genomic relationship matrices (GRMs) for genomic prediction in three different beef cattle breeds to identify the best approach for enhancing the accuracy of multi-breed genomic selection in beef cattle. Additionally, a comparison was conducted to evaluate the predictive precision of different marker densities and genetic correlations among the three breeds of beef cattle. The GRM between Yunling cattle (YL) and other breeds demonstrated modest affinity and highlighted a notable genetic concordance of 0.87 between Chinese Wagyu (WG) and Huaxi (HX) cattle. In the within-breed GS, BayesR demonstrated an advantage over GBLUP. The prediction accuracies for HX cattle using the BayesR model were 0.52 with BovineHD BeadChip data (HD) and 0.46 with whole-genome sequencing data (WGS). In comparison to the GBLUP model, the accuracy increased by 26.8% for HD data and 9.5% for WGS data. For WG and YL, BayesR doubled the within-breed prediction accuracy to 14.3% from 7.1%, outperforming GBLUP across both HD and WGS datasets. Moreover, analyzing multiple breeds using genomic selection showed that BayesR consistently outperformed GBLUP in terms of predictive accuracy, especially when using WGS. For instance, in a mixed reference population of HX and WG, BayesR achieved a significant accuracy of 0.53 using WGS for HX, which was a substantial enhancement over the accuracies obtained with GBLUP models. The research further highlights the benefit of including various breeds in the reference group, leading to enhanced accuracy in predictions and emphasizing the importance of comprehensive genomic selection methods. Our research findings indicate that BayesR exhibits superior performance compared to GBLUP in multi-breed genomic prediction accuracy, achieving a maximum improvement of 33.3%, especially in genetically diverse breeds. The improvement can be attributed to the effective utilization of higher single nucleotide polymorphism (SNP) marker density by BayesR, resulting in enhanced prediction accuracy. This evidence conclusively demonstrates the significant impact of BayesR on enhancing genomic predictions in diverse cattle populations, underscoring the crucial role of genetic relatedness in selection methodologies. In parallel, subsequent studies should focus on refining GRM and exploring alternative models for GP.

## 1. Introduction

Genomic selection (GS) is currently widely used in domesticated animal breeding [1]. The accuracy of genomic estimated breeding values (GEBV) is primarily contingent upon the magnitude of the reference population, thus rendering genomic evaluations predominantly applicable to breeds of substantial size [2]. In China, GS was introduced in Simmental beef cattle breeding in 2007. Yet, in many small local cattle populations with a large effective population size, it may be challenging or even impossible to gather a sufficiently large training population for accurate GEBV prediction. Combining animals from multiple breeds has been suggested as a strategy to address the limitation of small reference population sizes. Yet, this method has only had moderate success because of inconsistent connections between single nucleotide polymorphisms (SNPs) and quantitative trait loci (QTLs) in various breeds (populations), along with the varying linkage disequilibrium (LD) between SNPs and QTLs in populations with less genetic similarity [2,3,4,5,6].

Previous research utilizing SNP markers from the Illumina 50K SNP chip has indicated that the intervals between markers are excessively wide for maintaining a consistent LD phase across different breeds, resulting in the accuracies of cross-breed predictions being negligible [7,8,9]. The BovineHD BeadChip (HD chip; Illumina Inc., San Diego, CA, USA), which is a high-density genotyping array containing 777,609 SNPs, was created in 2010, as stated in the literature. This chip is known for its short-range linkage disequilibrium, which is anticipated to persist across different breeds of cattle [10]. Conversely, whole-genome sequencing (WGS), a method developed more recently, has been employed to encompass a broader array of genetic variants than is possible with SNP arrays. WGS data are fundamentally distinct from the information acquired through dense SNP chips, as WGS encompasses all types of genetic variants, including SNPs, indels, copy number variants (CNVs), and others. Given that WGS data capture all variants, both rare and common, within a population, they have the potential to offer more precise indicators for causative mutations, both within and across breeds. Therefore, it is possible for forecasts to utilize factors beyond the linkage disequilibrium between SNPs and QTL. Consequently, the integration of WGS data has the potential to enhance the precision of genomic predictions. In multi-breed prediction scenarios, the incorporation of WGS data may decrease the necessity for SNP–QTL associations, which can differ among the various breeds under evaluation [8]. Although Meuwissen reported an advantage of WGS data over dense SNP data using simulated data, their results were restricted to within-population predictions and to a small number of QTLs/morgan [11]. Therefore, the advantages of utilizing WGS data for predicting outcomes across various breeds and unique small populations remain inadequately understood.

Various methods for GP across multiple breeds have been created, including GBLUP [12,13,14] and Bayesian models [8,12,15]. The primary focus of research in multi-breed GBLUP models lies in the innovation of construction methods for the G-matrix. Zhou et al. utilized a 50k SNP chip in the analysis of genomic relationship matrices (GRM) for two breeds, incorporating various sources of information such as LD phase consistency and marker effects [16,17]. Their study indicated that incorporating LD phase consistencies, marker effects, or both into the two-breed GRM did not lead to enhanced accuracies in two-breed predictions. When comparing methods, these researchers confirmed the advantage of Bayesian approaches compared with genomic BLUP for EBV estimation [2,7,10]. They stated that setting a large proportion of SNP effects to zero is necessary to take advantage of the density of the HD chip [7]. This conclusion agreed with conserved QTL–marker associations at small distances only. Furthermore, adding a polygenic component avoids spurious SNP–QTL associations due to pedigree relationship [18] and helps to select QTLs with rare alleles, small effects, or both [19,20]. Inclusion of a polygenic component also increases the accuracy of GEBV prediction and allows for regression slopes closer to 1 [7,10].

The objective of this study was (1) to compare the prediction performance using Unweight G-Matrix GBLUP ($G_{UNW}$-GBLUP), LD Weight G-Matrix GBLUP ($G_{LD}$-GBLUP), and BayesR models for within-population and multi-breed reference populations; and (2) to assess how much predictive accuracy is gained by using WGS data compared to HD chip SNP data, with emphasis on divergent cattle breeds with different genetic correlations.

## 2. Material and Methods

### 2.1. Ethics Statement

We adhered to guidelines of the China Council on Animal Care, with all steps approved by the Institute of Animal Science and the Chinese Academy of Agricultural Science in Beijing. Animal examinations were conducted in accordance with China’s Animal Welfare Council regulations.

### 2.2. Animals and Phenotype

The real dataset included 1478 HUAXI cattle (HX), derived from Chinese Simmental beef cattle, 600 Chinese Wagyu cattle (WG), and 400 Yunling cattle (YL). The HX cattle, which were bred on five farms located in the Ulgai Grassland of the Xilingole League in Inner Mongolia, China, were born over a span of 12 years from 2008 to 2020. Following the weaning stage, the animals were relocated to Beijing for the purpose of fattening, where they were consistently raised under identical feeding conditions. The WG cattle originated from Dalian and Haikou cities in China, with birthdates ranging from 2012 to 2022. In contrast, the YL cattle were born between 2020 and 2023, hailing from Kunming city in China. The age at which the animals were slaughtered varied between 18 and 32 months. In this research, slaughter weight (SWT; kg) was analyzed. The SWT was measured prior to slaughter following a 24 h fasting period.

### 2.3. Genotyping Data

DNA was extracted from blood samples obtained from the three cattle breeds. Illumina BovineHD BeadChips were utilized for genotyping, containing a total of 777,962 SNPs. We conducted SNP quality control for each breed using PLINK v1.9 [21]. SNP genotypes for individuals were removed if the call rate (CR) was less than 90%, the minor allele frequency (MAF) was below 0.01, or if the SNP genotype frequency showed a significant deviation from the Hardy–Weinberg Equilibrium (*p* < 1.0 × 10^−6^). Only autosomal SNPs were included in the subsequent analyses. Subsequently, a total of 1478 individuals and 672,060 SNPs were retained and phased using BEAGLE v4.1 with default parameters, as outlined by Browning [22].

A total of 44 unrelated HX, 20 WG, and 20 YL cattle specimens were selected for the purpose of conducting genetic resequencing of the whole genome. Each animal sample was subjected to library preparation using the Illumina Hiseq 2500 genome sequencing system (Illumina Inc., San Diego, CA, USA). Each sample was sequenced about 20X on average with a range of 17X to 25X, with X representing the number of times each base was sequenced [23].

We obtained a total of 9,621,765,847 reads, which were subsequently subjected to our quality control procedures. After the imputation process, a total of 12,468,401 markers were obtained for chromosomes 1–29 among the breeds. A more detailed description was presented in our recent publication [23].

### 2.4. Genetic Correlations

We utilized the persistence of the LD phase between populations to determine the genetic relationship among the breeds. In this method, we calculated the correlation of linkage disequilibrium (r^2^) of adjacent marker pairs on each autosome and used the average correlation to represent the genetic relationship [24,25]. The calculation of r was proposed by Hill and Robertson [26], and r^2^ was calculated as:

$$r^{2}=\frac{D^{2}}{f(A)f(a)f(B)f(b)}$$

where $D=f\left(AB\right)-f\left(A\right)f(B)$, and $f\left(AB\right)$,$\;f\left(A\right)$, $f\left(a\right)$, $f\left(B\right)$, and $f\left(b\right)$ are the observed frequencies of haplotype *AB* and alleles *A*, *a*, *B*, and *b*, respectively.

### 2.5. Statistical Models

#### 2.5.1. GBLUP Model

The GBLUP method was used in this study to predict breeding values [27]. The model was given by:

y=Xb+Zg+e

where y is a vector of the phenotypes, X is a design matrix relating the fixed effects to each animal, b is a vector of fixed effects, **Z** is a design matrix allocating records to genetic values, g is a vector of additive genetic effects for an individual, and e is a vector of random normal deviates with variance $\sigma_{e}^{2}$. In addition, $var(g)=G\sigma_{g}^{2}$ where G is the genomic relationship matrix and $\sigma_{g}^{2}$ is the genetic variance for this model. The vector g contains animals with phenotypic data but can be extended to animals without phenotypes.

#### 2.5.2. Multi-Breed GBLUP

The same trait from different breeds was regarded as two different traits in the multi-trait GBLUP model [17]. The model was given by:

$$\left(\begin{array}{c} y_{1}\\ y_{2} \end{array}\right)=\left(\begin{array}{c} {1\mu }_{1}\\ {1\mu }_{2} \end{array}\right)+\left(\begin{array}{cc} Z_{1} & 0\\ 0 & Z_{2} \end{array}\right)\left(\begin{array}{c} a_{1}\\ a_{2} \end{array}\right)+\left(\begin{array}{c} e_{1}\\ e_{2} \end{array}\right)$$

where $y_{1}$ was the vector of the phenotype of breed 1 and $y_{2}$ was the vector of the phenotype of breed 2. The variables $\mu_{1}$ and $\mu_{2}$ represent the respective means of the two breeds, vectors $a_{1}$ and $a_{2}$ represent the genomic breeding values, vectors $e_{1}$ and $e_{2}$ represent the residual effects, and $Z_{1}$ and $Z_{2}$ represent incidence matrices associating genomic breeding values with $y_{1}$ and $y_{2}$, respectively. For a detailed description of the model assumptions, please refer to Zhou et al. [17].

#### 2.5.3. Unweight G-Matrix ($G_{UNW}$)

The ***G***-matrix was constructed with four parts alone in the $G_{UNW}$ [28]. Two blocks were constructed within a breed for the purpose of analyzing the G-matrix, with identical construction methods used for each block. However, allele frequencies were computed separately for each individual breed. The between-breed blocks were created through a mathematical process involving the multiplication of genotypes from two different breeds and subsequent division by the geometric mean of the sum of 2p(1−p) values from each breed. This process allowed for the calculation of the genomic relationship coefficient ${(g}_{ij})$ between individual i from breed 1 and individual j from breed 2, as given by:

$$g_{ij}=\frac{\sum_{k=1}^{m}M_{1(i,k)}M_{2(j,k)}}{\sqrt{\sum 2p_{1,k}(1-p_{1,k})\sum 2p_{2,k}(1-p_{2,k})}}$$

where $M_{1}$ and $M_{2}$ represent the genotype matrices of breed 1 and breed 2, respectively; *m* is the total number of markers; and $p_{1,k}$ and $p_{2,k}$ denote the observed allele frequencies of $A_{2}$ at locus *k* for breed 1 and breed 2, respectively.

#### 2.5.4. LD Weight G-Matrix ($G_{LD}$)

The correlation of SNP effects between breeds can vary across the genome, so it is not valid to assume that the covariance is consistent for all SNPs [17]. We considered LD phase consistency when creating the two-breed GRM. Within-breed blocks of the $G_{LD}$ matrices were constructed without weighting, while differential marker weighting was only applied to between-breed blocks. For example, $g_{ij}$ of individual *i* in breed 1 and individual *j* in breed 2 was calculated as:

$$g_{ij}=\frac{\sum_{k=1}^{m}M_{1(i,k)}M_{2(j,k)}w_{k}}{\sqrt{\sum 2p_{1,k}(1-p_{1,k})\sum 2p_{2,k}(1-p_{2,k})}}$$

where $w_{k}$ represents the weight on marker *k*. LD phase consistencies were measured as correlations of all pairwise $r_{LD}^{2}$ between two breeds (indicated as ${cor}_{LD}$). The $r_{LD}^{2}$ measured LD between any pair of markers within a marker interval. We divided all SNPs (common to both breeds) into three intervals of different sizes, which contained 10 SNPs in each interval. We used these makers in each interval to calculate the LD phase consistencies (${cor}_{LD}$) and took $r_{LD}^{2}$ as the weight for all of the SNPs in the corresponding interval. For each locus, we calculated the $r_{LD}^{2}$ of the ten nearest loci and calculated the correlation of $r_{LD}^{2}$ between two breeds as weights. If there was only one shared pair, we used the mean of all SNP pairs instead. $M_{1}$, $M_{2}$, *m*, $p_{1,k},$ and $p_{2,k}$ were the same as above.

#### 2.5.5. BayesR Model

We used the hybrid variant of the BayesR model [29] that first used an expectation-maximization (EM) module, followed by a Monte Carlo Markov chain (MCMC) model for 10,000 iterations, fitting the following model:

$$y_{i}=Xb+\sum_{j=1}^{p}m_{ij}\alpha_{j}+Wv+e_{i}$$

where $y_{i}$ is a vector of the phenotypes; $m_{ij}$ is the genotype covariate at locus j for individual *i* (coded as 0, 1, 2); *p* is the number of genotyped loci; $\alpha_{j}$ is a vector of allele substitution effects for locus *j*; W is a design matrix of genotypes; and v a vector of variant effects, drawn from 4 normal distributions with N (0,0*$\sigma_{g}^{2}$), N (0,0.0001*$\sigma_{g}^{2}$), N (0,0.001*$\sigma_{g}^{2}$), and N (0,0.01*$\sigma_{g}^{2}$) where $\sigma_{g}^{2}$ is the additive genetic variance and prior distribution of the proportion of variants per distribution **P** ~ Dirichlet(α), with α = [1,1,1,1]; and $e_{i}$ is a vector of random residuals for individual *i*. Fixed effects or general mean, in this case, were assigned flat priors [1,30]. Fix effects and covariates, including gender, farm, breed, birth year, and individual age of slaughter (day), were considered in the model.

The results of each model were evaluated using the accuracy of predictions. The prediction accuracy ($r_{GEBV,\hat{y}}$) was measured with Pearson’s correlation coefficient, calculated as${\;r}_{GEBV,\hat{y}}=Cor(GBEV,\hat{y})$ where $\hat{y}\;$is the residual value after correcting the fixed effect. Each dataset was randomly divided into five groups, with four groups used as a training dataset and the remaining group used for validation, and 10 replicates of 5-fold cross-validation were employed for each trait. Accuracy was determined as the mean of the results for five-fold cross-validation procedures. We sampled 100 random individuals to compose the training population, and the remaining individuals composed the reference population [31].

## 3. Results

### 3.1. Descriptive Statistics of the Analyzed Traits

Table 1 presents a comprehensive summary of the statistical attributes of slaughter weights across three distinct cattle breeds: Huaxi cattle (${SWT}_{HX}$), Chinese Wagyu cattle (${SWT}_{WG}$), and Yunling cattle (${SWT}_{YL}$). WG had the highest average slaughter weights at 605.10 kg, a phenomenon that could potentially be attributed to the advanced age at which WG cattle were typically slaughtered. By contrast, YL cattle exhibited the lowest live weights prior to slaughter, measuring only 372 kg. The coefficient of variation ranged from 9.68% to 14.88% across the three breeds, suggesting a relative dispersion of data points. The limited phenotypic variation within each population indicated a high quality of phenotype data for the three breeds. Heritability estimates were 0.40 for ${SWT}_{HX}$, 0.53 for ${SWT}_{WG}$, and 0.49 for ${SWT}_{YL}$, suggesting a moderate to high genetic influence on these traits.

### 3.2. Genomic Relationship of Two Breeds

**Figure 1:**
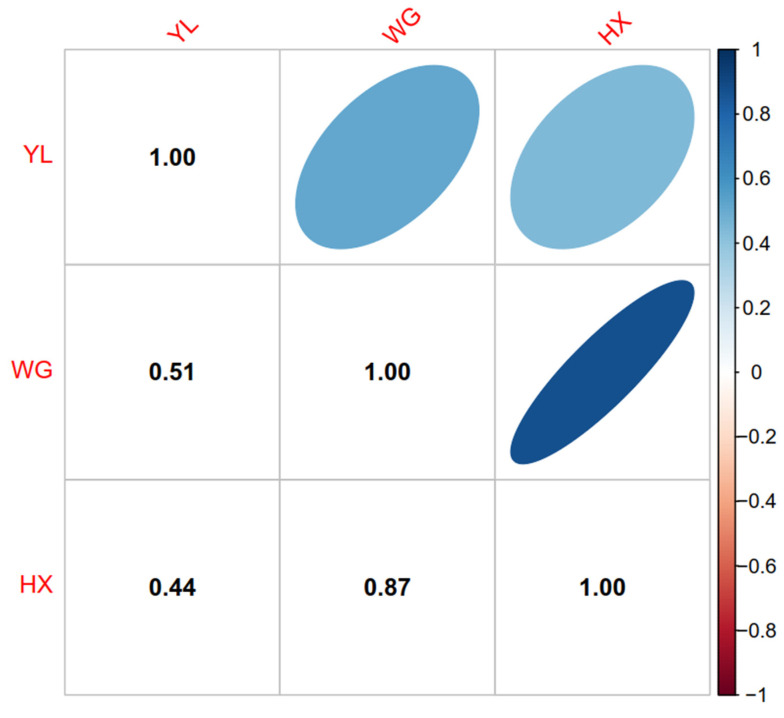
Genomic relationship coefficients of Huaxi cattle (HX), Chinese Wagyu cattle (WG), and Yunling cattle (YL).

Figure 1 illustrates the genetic relationship among the three cattle breeds: YL, WG, and HX. The off-diagonal elements represent the genetic relationships between breeds. The genetic similarity between YL and WG was represented as 0.51, between YL and HXN as 0.44, and between WG and HX as 0.87. These values suggested a moderate genetic relationship between YL and the other two breeds, and a high genetic similarity between WG and HX. It was calculated based on the persistence of the LD phase between populations to determine the genetic relationship among breeds. The values ranged from −1 to 1, where 1 indicates identical genetic makeup and values approaching 0 indicate lower genetic similarity. Furthermore, Figure S1 and Figure S2 respectively show the LD decay trends of the three breeds and the results of PCA cluster analysis, which to some extent indicate the kinship among breeds, thus verifying the reliability of the results in Figure 1.

### 3.3. Prediction Accuracy of Within-Breed GS

Table 2 compares the prediction accuracy of the two genomic selection methods, GBLUP and BayesR, using high-density chip data (HD) and whole-genome sequencing data (WGS) across the three cattle breeds: Huaxi (HX), Chinese Wagyu (WG), and Yunling (YL).

The BayesR model demonstrated prediction accuracies of 0.52 and 0.46 for HX cattle using BovineHD BeadChip data (HD) and whole-genome sequencing data (WGS), respectively. Compared to the GBLUP model, the BayesR model exhibited increases in accuracy of 26.8% for HD data and 9.5% for WGS data. Additionally, BayesR doubled the within-breed prediction accuracy for WG and YL cattle to 14.3% from 7.1%, surpassing the performance of GBLUP across both HD and WGS datasets. For Chinese Wagyu cattle, both GBLUP and BayesR showed equal accuracies of 0.34 (± 0.017 and ± 0.012, respectively) with HD. Similarly, with WGS, both methods yielded an accuracy of 0.38, but with slightly different standard errors (GBLUP: ± 0.012, BayesR: ± 0.011). In Yunling cattle, GBLUP demonstrated accuracies of 0.28 (± 0.021) with HD and 0.28 (± 0.019) with WGS. BayesR showed a slightly better performance, with accuracies of 0.30 (± 0.016) for HD and 0.32 (± 0.011) for WGS.

The table demonstrated that BayesR generally provided higher prediction accuracies compared to GBLUP, especially in the Huaxi cattle breed. The impact of SNP density (HD vs. WGS) varied depending on the breed and the method, with notable differences in Huaxi cattle but less so in the other breeds.

### 3.4. Accuracy of Multi-Breed GS

Table 3 presents the prediction accuracies of $G_{UNW}$-GBLUP, $G_{LD}$-GBLUP, and BayesR models using high-density chip data (HD) and whole-genome sequencing data (WGS) in various combinations of reference populations (refPop) for HX, WG, and YL cattle breeds.

The results highlighted significant differences in prediction accuracies between the GBLUP and BayesR models across different breeds and genomic data types. Notably, BayesR generally exhibited higher prediction accuracies than GBLUP models. For instance, in the HX + WG refPop, BayesR achieved prediction accuracies of 0.48 ± 0.014 with HD and 0.53 ± 0.012 with WGS in HX cattle, outperforming the GBLUP models. Similarly, across different refPop combinations, BayesR consistently demonstrates superior performance in capturing the genetic architecture of the traits, particularly with WGS data. Meanwhile, the average prediction accuracy of $G_{UNW}-GBLUP$ in the multi-breed strategy was similar to the within-breed strategy, and the prediction accuracy of $G_{LD}-GBLUP$ was usually higher than that of $G_{UNW}-GBLUP$ in the multi-breed and within-breed strategies.

Furthermore, the table also underscored the impact of different reference populations on prediction accuracy. The inclusion of multiple breeds in the reference population generally enhanced the prediction accuracy for each breed, indicating the benefit of multi-breed genomic selection approaches in capturing broader genetic variation.

## 4. Discussion

### 4.1. Impact of BayesR and GBLUP on GEBV in Single and Multiple Breed Genomic Selection

The study’s results indicated that BayesR outperformed GBLUP in genomic selection (GS), particularly in multi-breed scenarios involving Huaxi (HX), Wagyu (WG), and Yuling (YL) cattle breeds. The ability of BayesR to handle polygenic traits and complex genetic architectures led to higher prediction accuracies compared to GBLUP. This superiority was most evident in Huaxi cattle, where BayesR achieved prediction accuracies of 0.52 with high-density (HD) chip data and 0.46 with whole-genome sequencing (WGS) data, significantly exceeding those of GBLUP. These findings aligned with those of Wang et al. (Wang et al. 2016) and Irene et al. (van den Berg et al. 2017), who showed that the accuracy with the hybrid model was equal to that with BayesR, which confirmed that the hybrid model is an efficient alternative to BayesR. This superiority is particularly evident in multi-breed genomic selection scenarios, highlighting the potential of BayesR in handling the complex genetic architecture of diverse cattle populations.

The efficacy of genomic predictions utilizing GBLUP is contingent upon the magnitude of the reference population [20,32]. Consequently, when a substantial reference population was accessible for a singular breed of HX, GBLUP attained a significant portion of the prospective accuracy for genomic predictions, thereby rendering the utilization of nonlinear BayesR techniques for prediction or WGS genomic markers seemingly inconsequential [9]. However, Habier et al. [33] indicated that the efficacy of genomic prediction using GBLUP with medium-density SNPs diminishes when more robust predictions are required, particularly for predicting the genetic merit of distantly-related animals, such as those in future generations or from different breeds. They further reported the inferior predictive performance of GBLUP over successive generations in comparison to BayesB [33]. Nevertheless, our findings demonstrate the merits of employing nonlinear genomic prediction methods for multi-breed predictions.

### 4.2. Effect of Using LD Information Weighted GRM on GEBV Accuracy

In our findings, it was observed that the prediction accuracy of $G_{LD}$-GBLUP generally slightly surpassed that of $G_{UNW}$-GBLUP when employing both multi-breed and within-breed strategies. The use of LD information in weighting GRMs can impact the accuracy of GEBV [34]. LD-weighted matrices capture the non-uniform distribution of informative markers across the genome, which is crucial for traits influenced by regions with high LD. Such weighting schemes can enhance the prediction ability to capture the genetic architecture of traits, thereby improving prediction accuracy. This approach is particularly beneficial in populations with varying marker density and LD patterns, enabling more precise estimation of genetic similarities and differences.

However, concurrently, certain articles present an alternative perspective. The study conducted by Zhou et al. revealed that the inclusion of LD phase consistencies, marker effects, or a combination of both in the weighting of the G-matrices in Nordic Holstein and Nordic Red cattle did not result in any notable improvement in the accuracies of the two-breed predictions [17].

### 4.3. Influence of Genetic Correlation between Different Breeds on GEBV Accuracy

Variations in complex traits across populations can be attributed to disparities in genetic and environmental factors [28]. In the current study, the application of LD phase persistence in determining relationships among breeds demonstrated the impact of LD-weighted GRMs on GEBV accuracy. This underscores the importance of considering LD information in GRM construction, especially in multi-breed genomic selection scenarios [25].

The examination of genetic correlations among populations offers valuable insight into the variations in genetic architecture of traits across different populations [35]. This is pivotal for genomic prediction strategies, as evidenced by Lund et al. and Brøndum et al., who showed improved prediction accuracy using closely related breeds in reference groups [3,35]. The study reiterates the necessity of considering genetic correlation and relatedness between populations when transferring genomic information across breeds. A diminished genetic correlation between populations suggests that causal loci exhibit distinct effects and/or that diverse causal loci underlie the trait [28].

Furthermore, genetic correlations offer valuable insight into the feasibility of utilizing data from multiple populations for the purpose of genomic prediction. In cases where the genetic correlation is low, the efficacy of merging populations into a single training population or incorporating information regarding the distribution of causal variants across populations, as practiced in multi-task Bayesian models [36,37], is unlikely to enhance the accuracy of estimated genetic values. This is primarily due to the expected dissimilarities in effects and locations of causal loci.

To carry out genomic prediction across multiple populations, it is not necessary to have a detailed and precise understanding of genetic (co)variances and correlations. Thus, the precision of estimated genetic values remains fairly uniform across different techniques for computing the GRM [38,39,40]. However, for forecasting accuracy in such scenarios, it is crucial to have an accurate estimation of genetic correlations [41]. In academic contexts, the amalgamation of populations is advantageous when the training population for one of the groups is limited in size and there exists a substantial degree of genetic relatedness and correlation between the populations. This circumstance is particularly evident in the scenario of subpopulations originating from the same breed but residing in disparate environments.

### 4.4. Impact of Marker Density on the Accuracy of Genomic Estimated Breeding Values

The study also addresses the influence of marker density on the accuracy of genomic predictions, highlighting that increased marker density enhances GEBV accuracy both in within- and multi-breed GS. This aligns with the understanding that higher marker density captures more genetic variation, providing a more detailed genetic profile for selection. This is particularly significant in multi-breed genomic selection contexts.

Irene et al. [15] demonstrated that the utilization of simulated data revealed superior accuracy in the analysis of sequencing data compared to the analysis of HD SNP genotypes. Specifically, the employment of WGS data conferred a significant advantage over the HD dataset, thereby leading to higher accuracy in all scenarios involving sequencing data as opposed to the HD dataset. The authors utilized authentic data to demonstrate that the superiority of WGS over HD genotyping was significantly less pronounced in the Red Holstein validation population compared to the Australian Red validation population. This discrepancy can be attributed to the conservation of LD over shorter distances across breeds compared to within breeds. Consequently, the utilization of sequencing data is believed to offer advantages in multi-breed and across-breed prediction scenarios [42].

There are two factors contributing to the enhanced accuracy achieved through the utilization of sequencing data: firstly, it has the potential to encompass a greater extent of genetic variability, and secondly, it can encompass QTLs with substantial effects in cases where there is an absence of high-density SNPs in complete LD with said QTLs. Irene et al. suggested that predictive models based on sequencing data should prioritize variants that are in closer proximity to the QTL, or even coincide with the QTL itself, as this LD is more effectively preserved in the validation population. Furthermore, the adoption of sequencing data serves to mitigate the issue of missing heritability [15].

## 5. Conclusions

The research findings indicate that BayesR outperforms GBLUP in multi-breed genomic predictions, particularly for breeds with divergent genetic backgrounds. Enhanced SNP marker density enhances the accuracy of these predictions. The study demonstrates that incorporating genetic relationships into genomic prediction models enhances prediction accuracy in cattle populations. Further investigation is warranted to refine genomic prediction models and enhance breeding strategies.

## Figures and Tables

**Table 1:** Summary statistics of the analyzed traits.

Trait	Number	Mean	SD	C.V	Minimum	Maximum	Heritability
${SWT}_{HX}$	1302	544.05	80.95	14.88	318	790	0.40 (0.04)
${SWT}_{WG}$	600	605.10	72.05	11.91	594	1001	0.53 (0.06)
${SWT}_{YL}$	400	372	36.01	9.68	295	420	0.49 (0.07)

Note: ${SWT}_{HX}$, slaughter weight for Huaxi cattle, kg; ${SWT}_{WG}$, slaughter weight for Chinese Wagyu cattle, kg; ${SWT}_{YL}$, slaughter weight for Yunling cattle, kg; C.V: coefficient of variation, %.

**Table 2:** Prediction accuracies of different methods for within-breed genomic selection.

refPop	Method	SNP Density	
		HD	WGS
HX	GBLUP	0.41 (0.013)	0.42 (0.010)
	BayesR	0.52 (0.010)	0.46 (0.008)
WG	GBLUP	0.34 (0.017)	0.34 (0.012)
	BayesR	0.38 (0.010)	0.38 (0.011)
YL	GBLUP	0.28 (0.021)	0.28 (0.019)
	BayesR	0.30 (0.016)	0.32 (0.011)

Note: refPop, reference population; HD, high-density chip data; WGS, whole-genome sequencing data; HX, Huaxi cattle; WG, Chinese Wagyu cattle; YL, Yunling cattle. The standard error of prediction accuracy is in parentheses.

**Table 3:** Prediction accuracies of different methods for multi-breed genomic selection.

refPop	Method	HX		WG		YL	
		HD	WGS	HD	WGS	HD	WGS
HX + WG	$G_{UNW}$-GBLUP	0.41 (0.014)	0.44 (0.013)	0.34 (0.014)	0.35 (0.015)	0.10 (0.014)	0.11 (0.013)
	$G_{LD}$-GBLUP	0.42 (0.015)	0.46 (0.016)	0.35 (0.013)	0.38 (0.013)	0.10 (0.014)	0.12 (0.012)
	BayesR	0.48 (0.014)	0.53 (0.012)	0.4 (0.016)	0.44 (0.014)	0.13 (0.019)	0.17 (0.016)
HX + YL	$G_{UNW}$-GBLUP	0.41 (0.015)	0.44 (0.017)	0.13 (0.015)	0.15 (0.013)	0.3 (0.011)	0.31 (0.015)
	$G_{LD}$-GBLUP	0.41 (0.019)	0.45 (0.018)	0.13 (0.019)	0.16 (0.015)	0.32 (0.015)	0.34 (0.019)
	BayesR	0.43 (0.016)	0.48 (0.013)	0.15 (0.014)	0.19 (0.012)	0.33 (0.016)	0.38 (0.011)
WG + YL	$G_{UNW}$-GBLUP	0.11 (0.018)	0.12 (0.017)	0.34 (0.015)	0.35 (0.018)	0.31 (0.017)	0.32 (0.017)
	$G_{LD}$-GBLUP	0.13 (0.017)	0.15 (0.015)	0.35 (0.015)	0.37 (0.017)	0.33 (0.013)	0.35 (0.011)
	BayesR	0.18 (0.016)	0.2 (0.013)	0.41 (0.018)	0.44 (0.018)	0.34 (0.016)	0.38 (0.014)
HX + WG + YL	$G_{UNW}$-GBLUP	0.42 (0.013)	0.44 (0.017)	0.35 (0.014)	0.36 (0.016)	0.31 (0.012)	0.32 (0.018)
	$G_{LD}$-GBLUP	0.42 (0.019)	0.45 (0.017)	0.36 (0.016)	0.4 (0.013)	0.32 (0.017)	0.34 (0.016)
	BayesR	0.45 (0.012)	0.49 (0.012)	0.43 (0.018)	0.48 (0.015)	0.34 (0.018)	0.39 (0.013)

Note: refPop, reference population; HD, high-density chip data; WGS, whole-genome sequencing data; HX, Huaxi cattle; WG, Chinese Wagyu cattle; YL, Yunling cattle. The standard error of prediction accuracy is in parentheses.

## Data Availability

The data used in this manuscript can be obtained by contacting the corresponding author.

## Supplementary Materials

The following supporting information can be downloaded at: https://www.mdpi.com/article/10.3390/genes15020253/s1, Figure S1. Plot of linkage disequilibrium (LD, $r^{2}$) against distance between SNPs in Huaxi cattle (HX), Chinese Wagyu cattle (WG), and Yunling cattle (YL) genomes. Solid curves show the expected decay of LD in the genome-wide data, as well as within and outside the divergence regions, estimated by nonlinear regression of $r^{2}$. Figure S2. Principal component analysis with first and second components (PCs) with the three different breeds.

## Abbreviations

CNV	Copy number variant
CR	Call rate
EM	Expectation-maximization
GBLUP	Genome-enabled best linear unbiased prediction
GEBV	Genomic estimated breeding value
GP	Genomic prediction
GRM	Genomic relationship matrices
GS	Genomic selection
HD	High density
HX	Huaxi cattle
HW	Hardy–Weinberg Equilibrium
LD	Linkage disequilibrium
MAF	Minor allele frequencies
MCMC	Markov chain Monte Carlo
PCV	Phenotypic coefficient of variation
QTL	Quantitative trait locus
SNP	Single nucleotide polymorphism
SWT	Slaughter weight
WG	Chinese Wagyu cattle
WGS	Whole-genome sequencing
YL	Yunling cattle
