# Supplementary figures and images for: Improving Genomic Predictions in Multi-Breed Cattle Populations: A Comparative Analysis of BayesR and GBLUP Models
Source: Genes (Basel). 2024 Feb 18;15(2):253. doi: 10.3390/genes15020253 (PMC10887749; doi:10.3390/genes15020253)

# LD decay

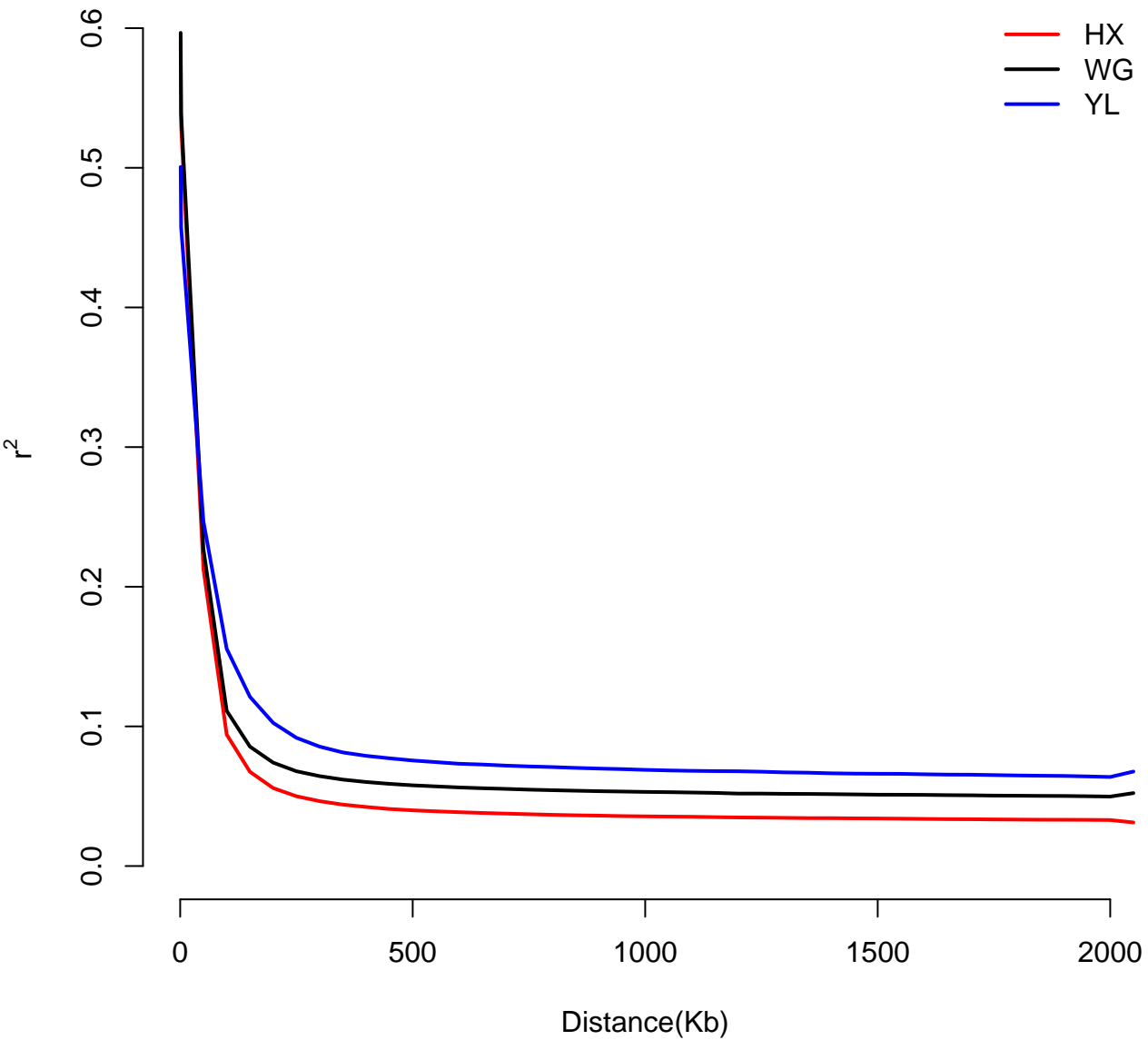

Supplement: Supplementary file 1 [file genes-15-00253-s001.zip › FigS1_LD.pdf]

PCA Plot

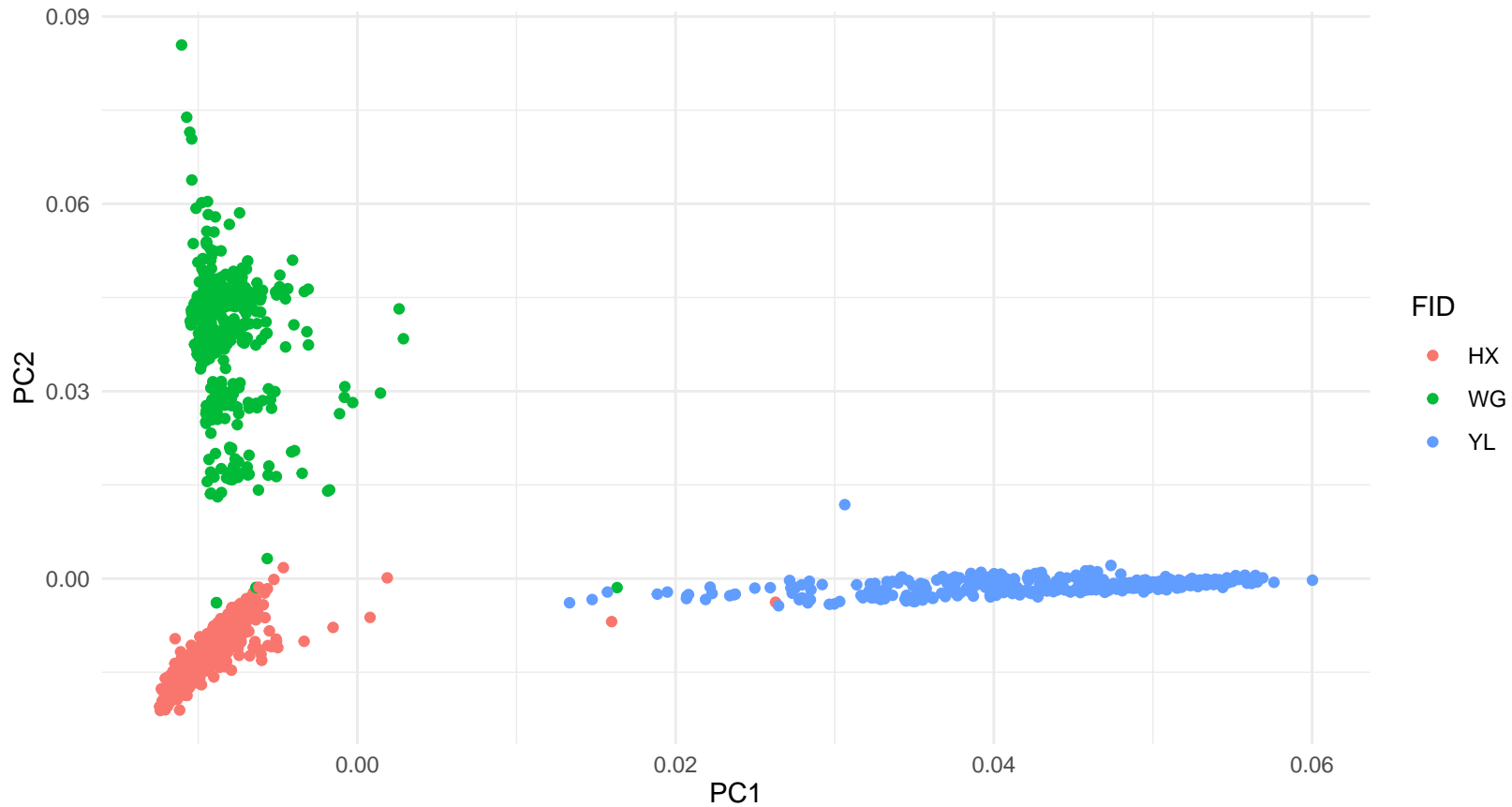

Supplement: Supplementary file 1 [file genes-15-00253-s001.zip › FigS2_PCA.pdf]
